# COVID-19: A New Challenge for Pulmonary Rehabilitation?

**DOI:** 10.3390/jcm10153361

**Published:** 2021-07-29

**Authors:** Magdalena Kołodziej, Justyna Wyszyńska, Monika Bal-Bocheńska

**Affiliations:** 1 Institute of Medical Sciences, Medical College of Rzeszow University, Kopisto 2a, 35-959 Rzeszow, Poland; 2 Institute of Health Sciences, Medical College of Rzeszow University, Kopisto 2a, 35-959 Rzeszow, Poland; justyna.wyszynska@onet.pl (J.W.); moniabb@vp.pl (M.B.-B.); 3 Podkarpackie Center for Lung Diseases in Rzeszów, Clinical Provincial Hospital No. 1, Rycerska 2a, 35-241 Rzeszow, Poland

**Keywords:** depression, coronavirus, pulmonary rehabilitation, respiratory function, SARS-CoV-2, quality of life

## Abstract

Coronavirus disease 2019 (COVID-19), currently one of the immense burdens for global healthcare, is often characterized by rapid progression and the occurrence of symptoms particularly affecting the respiratory system. Continuous refinement of treatment protocols improves prognosis; however, COVID-19 survivors are often left with the symptomatic burden of dyspnea and fatigue. Therefore, it is necessary to continue comprehensive treatment including pulmonary rehabilitation. This study aimed to review the available literature on pulmonary rehabilitation in patients diagnosed with COVID-19. The pulmonary rehabilitation programs implemented various forms, i.e., aerobic exercise, breathing exercises, effective cough exercises, diaphragmatic breathing, and respiratory muscle training. Based on the literature review, it was found that pulmonary rehabilitation programs result in an improvement of respiratory function, reduction of fatigue and dyspnea, and improvement in exercise endurance and quality of life after completing both short-term and long-term programs, but depression and anxiety problems did not improve. Pulmonary rehabilitation combined with psychological therapy is crucial for COVID-19 survivors and plays a substantial role in patients’ recovery.

## 1. Introduction

In late 2019, health professionals from China reported a new coronavirus, SARS-CoV-2, which occupied mainly the respiratory system [1]. In early 2020, Coronavirus disease 2019 (COVID-19) was declared a pandemic and thus scientists worldwide became focused on finding efficient treatment including the development of effective pulmonary rehabilitation programs [2]. COVID-19 survivors often report post-covid complications such as shortness of breath, fatigue, muscle pain, and weakness. Moreover, it was discovered that severe coronavirus pneumonia is associated with abnormal mucus production in the form of viscous and bloody secretions in airways caused by systematic inflammatory cytokine storm [3,4]. Consequently, respiratory functions are disturbed by lung fibrosis, airflow reduction, and respiratory muscle fatigue; therefore, the necessity of comprehensive pulmonary rehabilitation applied together with pharmacotherapy is indisputable.

Many health professionals and researchers reported rehabilitation protocols, consensus statements, and therapeutic interventions regarding pulmonary physiotherapy [5]. Some of them have prepared ready-to-use procedures, others discussed the necessity of pulmonary rehabilitation, and a few have reported quantitative results of physiotherapy effectiveness assessments [6,7,8,9,10].

The aim of this paper was to perform a literature review on the role of pulmonary rehabilitation in mild-to-severe COVID-19 patients and summarize the impact of applied programs on respiratory function, exercise capacity, mobility, quality of life, activities of daily living, anxiety, and depression.

## 2. Review of the Literature

### 2.1. The Applied Pulmonary Rehabilitation Methods

The pulmonary rehabilitation programs presented by Pancera et al. and Sakai et al. were inpatient with an inpatient remote program implemented additionally by Sakai et al. [7,9]. Liu et al. introduced an outpatient program, while Zha et al. and Wootton et al. discussed tele-rehabilitation feasibility [6,8,10].

For seven patients, early rehabilitation began at the intensive care unit (ICU) and was continued in COVID wards. Eighteen patients underwent remote physiotherapy in the COVID unit, 32 were treated in rehabilitation units, 36 participated in ambulatory rehabilitation, and for 63 individuals, physiotherapy was applied through tele-rehabilitation. During the described interventions, all patients were alive and there were no reports of death after completing the protocols.

### 2.2. Frequency and Duration

The duration of all the presented rehabilitation programs varies from one to six weeks. Two manuscripts presented the effectiveness of six-week physiotherapy programs [6,10] and one publication reported the effectiveness of a four-week program with physiotherapy applied twice daily [10]. The remaining two manuscripts assessed the efficacy of individually adjusted rehabilitation programs that lasted from four days up to three weeks and were applied up to five times a week [7,9].

### 2.3. Implemented Programs

All programs implemented pulmonary rehabilitation in various forms: aerobic exercise [7,9,10], breathing exercises including pursed-lip breathing, effective cough exercises, diaphragmatic breathing, and respiratory muscle training [6]. In addition, one publication presented traditional Chinese martial arts, the Eight Section Brocade, combining breathing, acupressure, and whole-body exercises [8].

Additionally, three programs included limb muscle strengthening exercises [7,9,10] and one included the activities of daily living training [7].

### 2.4. Pulmonary Physiotherapy in COVID-19: Summary of the Results

#### 2.4.1. Respiratory Function, Fatigue, and Dyspnea

Three investigators reported the impact on respiratory function. Liu et al. presented spirometry and diffusion capacity results: FEV1, FVC, FEV1/FVC ratio, and DLCO [6]. All spirometry values significantly increased but even after completing the six-week pulmonary rehabilitation program, the FEV1/FVC ratio was still below the lower limit (experimental group: 68.19 ± 6.05%; control group: 61.23 ± 6.43%). Diffusion capacity was also significantly improved in the experimental group and no change was observed in the control group.

Zha et al. assessed the impact of pulmonary rehabilitation based on self-reported respiratory symptoms such as dry or productive cough, difficulty in expectoration, and dyspnea. The symptoms reporting rate decreased three to four times after completing a modified rehabilitation program based on Chinese martial arts [8].

Wootton et al. evaluated oxygen saturation and heart rate at rest and during exercises, and to assess fatigue and dyspnea, they used the Fatigue Severity Scale (FSS) and modified Medical Research Council (mMRC) scales. No major improvement was found after completing the rehabilitation program but in contrast with other programs, no pulmonary exercises such as diaphragmatic breathing, respiratory muscle training, or cough exercises were applied [10].

#### 2.4.2. Exercise Capacity and Mobility

Exercise capacity was assessed in one study by a 6 min walking distance, showing statistically significant improvement after a six-week physiotherapy program focused on breathing exercises [6]. Mobility was evaluated in three studies by the Barthel Index Mobility (BIM) score [7], Short Physical Performance Battery (SPPB) [9], and Sit to Stand (STS) test [10]. The test results improved after completing both short-term (4–30 days) and long-term (six weeks) designed rehabilitation programs but statistical tests were not implemented. 

In addition, Pancera et al. showed a quadriceps girth increase after completing both short-term (1 week) and long-term (3 weeks) rehabilitation programs [9].

#### 2.4.3. Quality of Life

Quality of life (QoL) was assessed in two studies using the Short Form-36 (SF-36) health survey [6] and EuroQol questionnaire-5 dimensions, 3 levels (EQ-5D-3L) [9]. The implemented respiratory physiotherapy programs significantly improved all examined QoL aspects. In addition to the post-rehabilitation improvement, Liu et al. showed no changes in the control group compared to the experimental group [6].

#### 2.4.4. Activities of Daily Living

Three publications reported the impact of implemented rehabilitation programs on activities of daily living (ADL) using the Barthel Index total score [7,9] and Functional Independence Measure (FIM) scale [6]. All three studies showed significant improvement in all discussed ADLs.

#### 2.4.5. Anxiety and Depression

The study by Liu et al. additionally presented the impact of pulmonary rehabilitation on mental health using the Self-Rating Anxiety and Depression Scales (SAS and SDS, respectively) [6]. Anxiety levels decreased after completing the rehabilitation program but depression remained the same. There were no significant changes in these ratings in the control group.

The study design, aim, investigated population, rehabilitation protocols, outcome measurements, and results of reviewed studies are summarized in Table 1.

## 3. Summary

COVID-19 rehabilitation protocols, consensus statements, suggestions, and perspectives have been developed in many countries since the beginning of the pandemic. All physiotherapy suggestions are built on methods previously used in chronic respiratory conditions and on experience gained through the rapidly growing number of COVID-19 patients [11]. Currently applied methods encompass prone positioning, positive expiratory pressure (PEP) mask breathing, and low-intensity respiratory muscle training (RMT) for severe COVID-19. Mild and moderate COVID-19 physiotherapy recommendations contain medium-intensity RMT, diaphragmatic breathing, pursed-lip breathing, and endurance exercise training, with additional airway clearance techniques if productive cough occurs [4,12,13,14,15]. The above approach is suggested to be effective but a limited number of studies analyzed its efficacy.

The majority of the pulmonary physiotherapy programs implemented in the analyzed studies had positive effects on various health aspects affected by COVID-19. Nevertheless, all these programs need further discussion considering the impact on lung function, exercise capacity, and QoL is inconclusive.

Lung function, reported by Liu et al., was significantly improved by pulmonary physiotherapy and this is clearly visible in an almost two-fold increase in FVC values (pre 1.79 ± 0.52 L; post 2.36 ± 0.49 L). However, post-rehabilitation results of the FEV1/FVC ratio, indicating obstruction level, still remain below normal values (pre 60.48 ± 6.39 %; post 68.19 ± 6.05 %) [6]. The study by Wootton et al. showed that physiotherapy did not influence saturation and heart rate results but the physiotherapy program was mainly focused on whole-body exercise training [10]. The frequency of respiratory symptoms’ appearance, presented by Zha et al., was significantly reduced from 35–50% pre-rehabilitation to 5–15% post-rehabilitation [8]. All the above suggest that implementing comprehensive physiotherapy programs, especially those containing breathing exercises, are crucial for COVID-19 survivors but the application of longitudinal programs including intensive respiratory physiotherapy might be critical in managing pulmonary complications of COVID-19.

Only one study analyzed the impact of pulmonary physiotherapy on exercise capacity with a 6-min walk distance and the improvement in distance walked after the 6-week program was 49 m. The minimally significant difference for COVID-19 patients has not been established. However, for the moderate and severe chronic obstructive pulmonary disease (COPD) patients, it is expected to be between 25–35 m and thus we can suspect the resultant difference is clinically important [5,16,17,18]. Recently, standard 6-min walk distance modifications were suggested to improve sensitivity when applied in COVID-19 patients. To avoid false-positive results, a threshold value of 1400 feet should be the cut-off point of abnormality [19].

Mobility was assessed in three studies with three instruments: the BIM score, SPPB, and STS test [7,9,10]. The most noticeable improvement was observed for SPPB scores and STS values. The BIM scores improved from 5 to 10 in directly rehabilitated patients and no deterioration was noted in BIM scores in the remote rehabilitation group. All implemented tests presented improvement in mobility after completing designed pulmonary rehabilitation programs. However, statistics were not implemented and thus further studies are required to confirm this effect.

The QoL was appraised using two instruments: the SF-36 and EQ-5D-3L questionnaire [6,9]. In the study by Liu et al., the improvements in the experimental group were all statistically significant, whereas no difference was found in the control group. Anxiety levels as evaluated by the SAS scale also decreased significantly only in the experimental group. Interestingly, depression levels assessed by the SDS scale did not improve in any of the groups [6]. In the study by Pancera et al., all patients reported a significant reduction in mobility, self-care, usual activities, and pain issues but anxiety/depression remained on a moderate level [9]. As previously described, virus infections associated with prolonged isolation and quarantine led to sleep disorders, anxiety, and depression [20]. However, due to disinformation widespread in social media, exhausted health care workers, and medical equipment shortage, the COVID-19 pandemic might outweigh the previously known SARS and MERS epidemics [21]. Huang et al. described the long-term health consequences of patients with COVID-19. They found that depression or anxiety was reported among 23% of COVID-19 survivors six months after acute infection. Women had an OR 1.80 (CI: 1.39–2.34) for anxiety or depression compared with men [22]. Deng et al. reported depression in 48% of hospitalized COVID-19 patients, whereas Zarghami et al. found that 35% of COVID-19 outpatients experienced depression episodes [23,24]. The underlying mechanism of the psychiatric consequences of COVID-19 seems to be multifactorial and might include the direct effects of viral infection, the immunological response, corticosteroid therapy, ICU stay, social isolation, or stigma [22,23,24].

These findings suggest that pulmonary rehabilitation improves different QoL aspects and reduces anxiety associated with coronavirus but might be insufficient to address COVID-19-related depression. Further support and strategies to minimize the psychosocial consequences of COVID-19 after discharge should be considered.

Most of the reviewed pulmonary rehabilitation programs had a positive effect on lung function, exercise endurance, and QoL of patients with mild, moderate, and severe COVID-19. Nevertheless, the usefulness of these programs in everyday clinical practice requires further investigation with robust study designs and additional follow-ups.

## Figures and Tables

**Table 1:** Summary of all included studies.

Authors	Study Design	Study Aim	Population	Applied Rehabilitation Protocols	Rehabilitation Effects’ Measurements	Results
Liu et al. [6]	RCT	To investigate the effects of pulmonary rehabilitation on respiratory function, ADL, QoL, and psychological status of elderly patients with COVID-19	Experimental group: 36 patients with diagnosed COVID-19 24M, 12F 69.4 ± 8 y Control group: 36 patients with diagnosed COVID-19 25M, 11F 68.9 ± 7.6 y	Experimental group Setting: out-patient Duration: 6 weeks Frequency: 2 sessions per week Training components: - respiratory muscle training–hand-held resistance device (3 sets of 10 breaths) - cough exercises (3 sets of 10 active coughs) - diaphragmatic breathing (30 voluntary diaphragmatic contractions in the supine position with 1–3 kg weight as a resistance) - stretching exercise (respiratory muscle stretching in the supine position: move arms in flexion, horizontal extension, abduction, and external rotation) - home exercises (30 sets/d of pursed-lip breathing and cough) Control group: N/A	Respiratory function Exercise capacity: 6MWT ADL: FIM scale QoL: SF-36 scale Anxiety/depression: SAS/SDS scales	Experimental group: FEV1: pre 1.1 ± 0.08; post 1.44 ± 0.25; *p* < 0.05 *# FVC: Pre 1.79 ± 0.52; Post 2.36 ± 0.49; *p* < 0.05 *# FEV1/FVC%: Pre 60.48 ± 6.39; Post 68.19 ± 6.05; *p* < 0.05 *# DLCO%: Pre 60.3 ± 11.3; Post 78.1 ± 12.3; *p* < 0.05 *# 6MWT: Pre 162.7 ± 72; Post 212.3 ± 82.5; *p* < 0.05 *# FIM: Pre 109.2 ± 13; Post 109.4 ± 11.1; *p* > 0.05 SF-36: - Physical health: Pre 52.4 ± 6.2; Post 71.6 ± 7.6; *p* < 0.05 *# - Body role: Pre 61.2 ± 6.6; Post 75.9 ± 7.9; *p* < 0.05 *# - Physical pain: pre 63.5 ± 7.4; post 78.3 ± 7.8; *p* < 0.05 *# - General health: pre 61.8 ± 7.7; post 74.2 ± 7.9; *p* < 0.05 *# - Energy: pre 60.6 ± 6.9; post 75.6 ± 7.1; *p* < 0.05 *# - Social function: pre 59.4 ± 7.2; post 69.8 ± 6.4; *p* < 0.05 *# - Emotional role: pre 61.4 ± 6.9; post 75.7 ± 7; *p* < 0.05 *# - Mental health: pre 61.5 ± 6.2; post 73.7 ± 7.6; *p* < 0.05 *# SAS: pre 56.3 ± 8.1; post 47.4 ± 6.3; *p* < 0.05 *# SDS: pre 56.4 ± 7.9; post 54.5 ± 5.9 Control group FEV1: pre 1.13 ± 0.14; post 1.26 ± 0.32 FVC: pre 1.77 ± 0.64; post 2.08 ± 0.37 FEV1/FVC%: pre 60.44 ± 5.77; post 61.23 ± 6.43 DLCO%: pre 60.7 ± 12; post 63.0 ± 13.4 6MWT: Pre 155.7 ± 82.1; Post 157.2 ± 71.7 FIM: Pre 109.3 ± 10.7; Post 108.9 ± 10.1; SF-36: - Physical health: Pre 53.2 ± 7.7; Post 54.1 ± 7.5 - Body role: Pre 61.3 ± 7.2; Post 62 ± 7.3; - Physical pain: pre 63.5 ± 8.1; post 62.9 ± 7.9; - General health: pre 61.8 ± 8.4; post 61.4 ± 6.9 - Energy: pre 60.5 ± 7.1; post 61.2 ± 6.3; - Social function: pre 59.5 ± 7.0; post 58.9 ± 6.6; - Emotional role: pre 61.4 ± 7.4; post 60.8 ± 7.3 - Mental health: pre 61.6 ± 7.2; post 62.1 ± 7.6; SAS: pre 55.8 ± 7.4; post 54.9 ± 7.3 SDS: pre 55.9 ± 7.3; post 55.8 ± 7.1
Sakai et al. [7]	Observ.	To investigate the safety and effectiveness of remote rehabilitation for COVID-19 inpatients	Remote rehabilitation group: 18 patients with diagnosed COVID-19 12M, 6F 56 (21–70) y Direct rehabilitation group: 25 patients with diagnosed COVID-19 19M, 6F 72 (43–95) y	Setting: inpatient remote/direct rehabilitation Duration: remote 4–30 d; direct 4–27 d Frequency: remote 20 min session/d; direct 20 min session/twice per day Training components: - muscle exercises - aerobic exercises - ADL exercises (direct)	ADL: Barthel Index total score (BI) Mobility: Barthel Index Mobility score (BIM)	Remote rehabilitation group: BI: pre 90; post 90 BIM: pre 15; post 15 Direct rehabilitation group: BI: pre 40; post 70 BIM: pre 5; post 10
Zha et al. [8]	Observ.	Not clearly stated (presentation of the modified version of rehabilitation for COVID-19 patients)	60 patients with mild COVID-19 39M, 21F 54 (38–62) y	Setting: tele-rehabilitation Duration: 4 weeks Frequency: 2 sessions/d (session = 6–8 repetitions) Training components: modified rehabilitation exercises (MRE) based on Chinese martial arts Eight Section Brocade: (1) Overhead chest and shoulder stretch (1 set of 2 repetitions) (2) Standing heel rises and upper body acupressure (2 sets of 12 repetitions) (3) Upper body rotation (1 set of 4 repetitions) (4) Hand acupressure massage (3 sets of 12 repetitions)	Self-reported respiratory symptoms: Dry cough Productive cough Difficulty in expectoration Dyspnea	Dry cough: pre 41.7%; post 11.7% Productive cough: 43.3%; post 11.7% Difficulty in expectoration: pre 35%; post 5% Dyspnea: pre 50%; post 15%
Pancera et al. [9]	Case series	To investigate the feasibility of a subacute rehabilitation for COVID-19 patients	7 patients diagnosed with severe COVID-19 7M; 37–61 y	Setting: inpatient Duration: 1–3 weeks Frequency: 45 min/d, 5 d/week Training components: (1) Weaning from mechanical ventilation (30 min spontaneous breathing through heat and moisture exchanger + PEP + expiratory muscle training) (2) Pulmonary rehabilitation (cycle ergometer aerobic training 30 min, 20 watts, and increasing slowly during following sessions) (3) Physiotherapy (sit-to-stand training: 3 sets, 5–10 repetitions; walking; resistance training with elastic bands: max 3 sets of 10 repetitions)	ADL: Barthel Index total score (BI); Barthel Index based on Dyspnea (BID) QoL: EQ-5D-3L Mobility: SPPB Muscles: MRC sum score; quadriceps (Q) girth (L/R)	Patient 1: BI: pre 25; post 100; BID: pre 83; post 71; EQ-5D-3L: pre 33323; post 11111; SPPB: pre 0; post 12; MRC sum score: pre 47; post 57; Q girth: pre 38/39.5; post 44.5/45.5 Patient 2: N/A Patient 3: BI: pre 23; post 100; BID: pre 86; post 2; EQ-5D-3L: 33323; post 11111; SPPB: pre 0; post 12; MRC sum score: pre 49; post 56; Q girth: pre 37/39.5; post 43.5/44 Patient 4: BI: pre 28; post 100; BID: pre 83; post 2; EQ-5D-3L: pre 33323; post 11112; SPPB: pre 0; post 12; MRC sum score: pre 51; post 60; Q girth: pre 31.5/32; post 34/34.5 Patient 5: BI: pre 19; post 100; BID: pre 80; post 5; EQ-5D-3L: pre 33333; post 11112; SPPB: pre 0; post 12; MRC sum score: pre 52; post 58; Q girth: pre 40.5/40.5; post 44/44.5 Patient 6: BI: pre 30; post 100; BID: pre 78; post 0; EQ-5D-3L: pre 33322; post 11111; SPPB: pre 0; post 12; MRC sum score: pre 51; post 60; Q girth: pre 41/41.5; post 43.5/44 Patient 7: BI: pre 77; post 100; BID: pre 40; post 5; EQ-5D-3L: pre 22211; post 11111; SPPB: pre 8; post 12; MRC sum score: pre 54; post 60; Q girth: pre 42/43; post 45/45.5
Wootton at al. [10]	Case series	Not clearly stated (to present tele-rehabilitation model for COVID-19 patients)	3 patients with diagnosed moderate to severe COVID-19 3M; 59–80 y	Setting: tele-rehabilitation Duration: 6 weeks Frequency: 4–6 d/week Training components: (1) Aerobic exercise training (walking 5–30 min) (2) Intermittent exercises (2 min intervals, 1 min rest) (3) Strengthening exercises (sit-to-stand, wall push-ups, heel raises, bicep curls, tricep dips: 2 sets, 10 repetitions each)	Mobility: 5STS (sec), 1minSTS (repetitions) Fatigue and dyspnea: FSS; mMRC scale Cardiorespiratory functions: SpO2% (rest/exertion), HR (rest/exertion)	Patient 1: 5STS: pre 10.66; post 5.06; 1minSTS: pre 26; post 46; FSS: pre 9; post 9; mMRC: pre 0; post 0; SpO2: pre 96/96; post 98/99; HR: pre 82/103; post 71/105 Patient 2: 5STS: pre 11.48; post 8.45; 1minSTS: pre 27; post 32; FSS: pre 20; post 33; mMRC: pre 2; post 1; SpO2: pre 97/96; post 99/99; HR: pre 85/110; 80/129; Patient 3: 5STS: pre 18; post 13.18; 1minSTS: pre 18; post 22; FSS: pre 13; post 29; mMRC: pre 2; post 2; SpO2: pre 97/97; 98/98; HR: pre 75/83; post 72/85

Abbreviations: ADL = activities of daily living; d = day; DLCO = diffusion lung capacity for carbon monoxide; EQ-5D-3L = EuroQol questionnaire, 5 dimensions, 3 levels; F = female; FEV1 = forced expiratory volume in 1 second; FIM = Functional Independence Measure; FSS = Fatigue Severity Scale; FVC = forced vital capacity; HR = heart rate; M = male; MRC = Medical Research Council; N/A = not available; Observ. = observational study; PEP = positive expiratory pressure; RCT = randomized controlled trial; SAS = Self-reported Anxiety Scale; SDS = Self-reported Depression Scale; SF-36 = Short Form-36 questionnaire; SpO2 = oxygen saturation; SPPB = Short Physical Performance Battery; STS = sit-to-stand; Q = quadriceps; QoL = quality of life; y = years; and 6MWT = 6-min walking test.

## Data Availability

No new data were created or analyzed in this study. Data sharing is not applicable to this article.
